# Improvement of diagnostic yield in carbamoylphosphate synthetase 1 (*CPS1*) molecular genetic investigation by RNA sequencing

**DOI:** 10.1002/jmd2.12091

**Published:** 2020-01-09

**Authors:** Jasmine Isler, Véronique Rüfenacht, Corinne Gemperle, Gabriella Allegri, Johannes Häberle

**Affiliations:** ^1^ Division of Metabolism and Children's Research Center University Children's Hospital Zurich Zurich Switzerland

**Keywords:** carbamoylphosphate synthetase 1, CPS1, next‐generation sequencing, RNA analysis, urea cycle defects

## Abstract

Carbamoylphosphate synthetase 1 (CPS1) deficiency is a rare inborn error of metabolism leading often to neonatal onset hyperammonemia with coma and high mortality. The biochemical features of the disease are nonspecific and cannot distinguish this condition from other defects of the urea cycle, namely *N*‐acetylglutamate synthase deficiency. Therefore, molecular genetic investigation is required for confirmation of the disease, and nowadays this is done with increasing frequency applying next‐generation sequencing (NGS) techniques. Our laboratory has a long‐standing interest in *CPS1* molecular genetic investigation and receives samples from centers in Europe and many other countries. We perform RNA‐based *CPS1* molecular genetic investigation as first line investigation and wanted in this study to evaluate our experience with this approach as compared to NGS. In the past 15 years, 297 samples were analyzed, which were referred from 37 countries. CPS1 deficiency could be confirmed in 155 patients carrying 136 different genotypes with only a single mutation recurring more than two times. About 10% of the total 172 variants comprised complex changes (eg, intronic changes possibly affecting splicing, deletions, insertions, or deletions_insertions), which would have been partly missed if only NGS was done. Likewise, RNA analysis was crucial for correct interpretation of at least half of the complex mutations. This study gives highest sensitivity to RNA‐based *CPS1* molecular genetic investigation and underlines that NGS should be done together with copy number variation analysis. We propose that unclear cases should be investigated by RNA sequencing in addition, if this method is not used as the initial diagnostic procedure.

## 1 INTRODUCTION

Carbamoylphosphate synthetase 1 (CPS1, E.C. 6.3.4.16) catalyzes, as first and rate‐limiting reaction of the urea cycle, the entry of ammonia into the cycle. This enzyme is encoded by *CPS1* (MIM *608307), located on chromosome 2q35 and composed of 38 exons leading to a 1500 amino acid protein.^1^, ^2^, ^3^ Mutations in *CPS1* can result in reduced or absent enzyme function leading to hyperammonemia and other features of CPS1 deficiency such as neonatal or late onset encephalopathy with vomiting, seizures, and coma if left untreated (CPS1D, MIM #237300).^4^ Diagnosis of CPS1D is based on a biochemical profile with increased plasma ammonia, decreased plasma citrulline, and a normal or low orotic acid in urine. Confirmation of the diagnosis requires either enzyme analysis in liver or small intestinal tissue or, recommended as method of choice, molecular genetic investigation.^5^, ^6^

More than 230 *CPS1* mutations are currently reported.^7^, ^8^, ^9^, ^10^, ^11^, ^12^, ^13^, ^14^, ^15^ Molecular genetic investigation for CPS1D can use different methods: exon per exon sequencing (direct Sanger sequencing), next generation sequencing (NGS) as part of a (often custom‐made) gene panel or whole exome or whole genome sequencing with or without the analysis of copy number variation (CNV), and RNA analysis.^16^, ^17^, ^18^

While automated sequencing applying NGS became more widely available in recent years and showed improved detection rates if compared to direct Sanger sequencing,^19^ our laboratory performed in most cases RNA analysis for the confirmation of CPS1D.^6^, ^16^ Source of RNA can be liver tissue but also skin fibroblasts^17^ or, hereby improving the turn‐around time, peripheral lymphocytes that were stimulated with phytohemagglutinin hereby applying a straightforward and less invasive protocol.^16^ Advantage of the latter approach is its simplicity and an improved sensitivity since, for instance, deletions or insertions caused by intronic changes are picked up in addition as their effect on RNA is instantly observable.

In this study, we wanted to evaluate our experience in performing RNA‐based *CPS1* molecular genetic investigation over the past 15 years. To do so, we investigated all found *CPS1* variants in this period, identified with varying methods, and analyzed the sensitivity of the applied approaches. We hypothesized that RNA analysis would reduce the number of missed mutations if compared to direct Sanger sequencing, and would even be better than NGS techniques.

## 2 MATERIALS AND METHODS

From 2004 to 2018, a total of 297 samples were referred with a suspicion of CPS1D (analyses were done at the University Children's Hospital Münster, Germany, until 2008, and at the University Children's Hospital Zurich since 2008). From (index) patients, our laboratory received mainly cultured fibroblasts or heparin blood, but also EDTA blood for DNA isolation, DNA, dried blood spots, or shock‐frozen liver tissue. Parents' samples were used in case the index patient was deceased and no material was available, and/or for confirmation of obligate heterozygosity. From the referring letter, we tried to collect information on origin of patients, onset of disease, and severity of the clinical course. As molecular genetic investigation was part of clinical diagnostics, there was no requirement for an approval by the respective ethics committees.

Assuming that single nucleotide changes in exons or flanking intronic sequences would not pose a problem for neither of the sequencing methods, we focused our analysis on complex mutations including intronic changes possibly affecting splicing as well as deletions, insertions, and INDELs. Four different sequencing methods were considered: direct Sanger sequencing of DNA isolated from EDTA blood or dried blood spots, NGS without or with CNV, and RNA sequencing using liver, fibroblasts, or lymphocytes. We arbitrarily defined ±50 bp flanking the exons as limit for an in general “probably detected” change with however maybe uncertain interpretation. For analyzing splicing variants within these limits, softwares Human Splicing Finder and MutationTaster were used.^20^, ^21^

## 3 RESULTS

During the study period, 297 samples were sent from 37 countries in Europe, North and South America, Australia, Asia, and Africa. CPS1D could be confirmed in 155 patients (52 diagnosed in Münster and 103 in Zurich) from 33 countries; patients referred from France (n = 32) and Turkey (n = 27) comprised the largest groups of positive samples. For the confirmed cases, we received mainly fibroblasts (n = 79) or heparin blood (n = 57) for index patient testing, but also two liver biopsies. In 17 families, there was no sufficient material available from the index patient. Therefore, mutations were initially searched for in parents (16 heparin blood samples and 1 fibroblast cell line), and were, whenever possible, confirmed in DNA (often derived from dried blood spots) of the patient.

Clinical information was not always provided, but the majority of the patients showed a neonatal onset. No strict correlation was found between type of mutation and onset of disease,^11^ except for a deletion in exon 25 (c.3037_3039delGTG) that was always associated with a neonatal onset of severe disease.^22^ Of the total 155 patients, 83 were homozygous but clinical information was too scarce to further correlate this. The remaining 72 patients were compound heterozygous.

**Figure 1:**
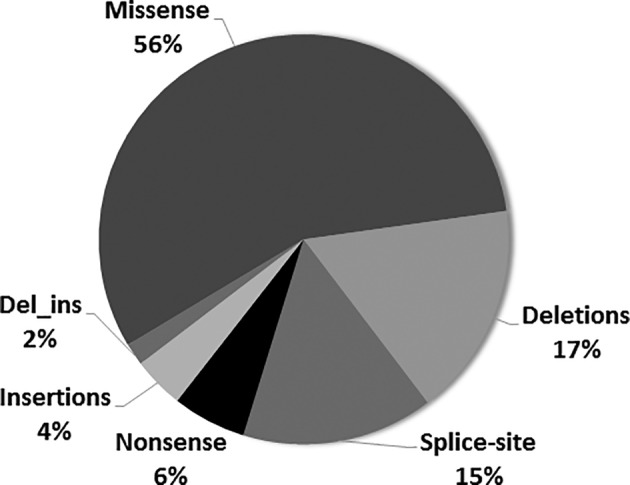
Graph showing the distribution of all *CPS1* mutations in this study

Underlining genetic heterogeneity at the *CPS1* locus, we identified a total of 136 different genotypes with 172 different variants, of which 56% were missense (n = 97), 17% deletions (n = 29), 15% splice‐site (n = 26), 6% nonsense (n = 10), 4% insertions (n = 7), and 2% INDELs (n = 3; Figure 1 and Table 1). Of the total 172 different variants, 31 missense, 2 nonsense, 13 splice‐errors, and 16 Del/Ins/Dup were not yet reported in literature (summarized in Tables S1 and S2).

**Table 1:** Summary of *CPS1* mutations of the study cohort

Item	#
Index patients	155
*Genotypes*	
Homozygous	64
Heterozygous‐compound	72
Total	136
Recurrent	1
*Mutations*	
Missense	97
Nonsense	10
Splice‐errors	26
Deletions	29
Insertions/duplications	7
Del_Ins	3
Total mutations	172
Recurrent	9

*Note*: Recurrence of genotypes or mutations in this study was defined as occurrence >2 times.

The most frequent mutation in this study, present in 17 Turkish patients, was the homozygous deletion in exon 25 c.3037_3039delGTG (p.Val1013del). Next, the missense mutation c.2339G>A (p.Arg780His) in exon 19 was found in seven patients (of varying ethnic background and nationality), of which two are homozygous for this change. The most frequent splice‐site mutation was c.3558+1G>C (p.Glu1161_Arg1186del) in exon 29, identified in three patients.

**Figure 2:**
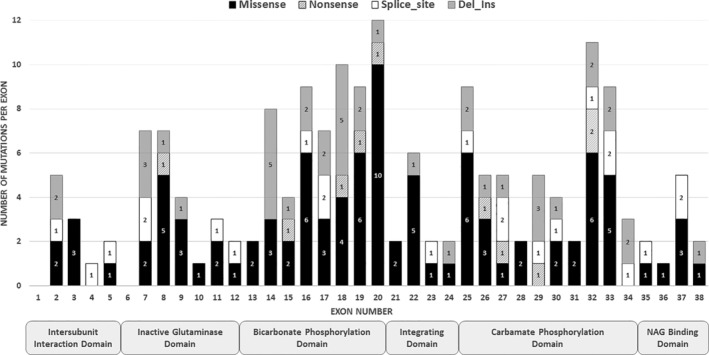
Distribution of mutations over the *CPS1* gene and its domains

As reported before,^11^ distribution of *CPS1* mutations shows predominance of the catalytic domains (116 of 172 mutations): the bicarbonate phosphorylation domain (exons 13‐20) was affected by 61 mutations and the carbamate phosphorylation domain (exons 25‐34) by 55 mutations (Figure 2).

Parental DNA could be investigated in 98 families confirming obligate carriership in 93 parents. In five families, only one of the parents was found to be a carrier, namely in three parents only the mother and in two parents only the father.

We identified and further analyzed 14 complex mutations (ie, intronic changes possibly affecting splicing, deletions, insertions, and INDELs), which were all found in single patients only and contributed to 14 different genotypes; within this subcohort, only three patients were homozygous. Details to the complex mutations in this study are summarized in Table 2. From these 14 complex mutations, using exon‐wise sequencing or NGS without CNV, five mutations would have been missed; using NGS even with CNV, two mutations would have been missed. RNA analysis did in fact identify all these mutations, and was necessary for a correct interpretation in half of the cases (Table 2).

**Table 2:** Summary of 14 complex mutations of the *CPS1* gene and their identification by different methods

Exon	Nucleotide	Protein	RNA	State in this study	Detection only with RNA analysis	Detection with NGS ± CNV	Possible misalignment error with NGS ± CNV	Correct interpretation only with RNA analysis	Human splicing finder	Mutation taster
2	c.127‐26_127‐24delinsCAG	p.(Ala43Aspfs*22)	r.127_128ins(23)	Heterozygous		x		x	Affecting splicing	Benign
7	c.622‐24A>G	p.(Asp208_Lys237del)	r.622_711del (Exon 7)	Homozygous		x		x	Benign	Benign
7	c.622‐52_c.711+1416del	p.(Asp208_Lys237del)	r.622_711del (Exon 7)	Heterozygous		x with CNV				
7	c.622‐7A>G	p.(Lys207_Asp208insTrpGln)	r.621_622insTGGCAG	Homozygous		x		x	Affecting splicing	Disease causing
8	c.712‐430_766del	p.(Arg238Metfs*5)	r.712_840del (Exon 8)	Heterozygous		x with CNV				
9	c.947G>T	p.(Arg316Metfs*2)	r.946_947insTGTGA	Heterozygous		x		x	Affecting splicing	Disease causing
15	c.1549+124_2391+800del	p.(Val518Hisfs*8)	r.1550_2391del (Exon 15‐19)	Homozygous		x^a^				
17	c.1837‐8A>G	p.(Ala613Phefs*25)	r.1836_1837insTTTCTAG	Heterozygous		x		x	Affecting splicing	Disease causing
18	c.2079_2080ins CATTCATTCATTCATT	p.(Val694Hisfs*8)	r.2079_2080ins CATTCATTCATTCATT	Heterozygous			x			
24	c.2895+429_c.2960‐281del	p.(Glu966Alafs*27)	r.2896_2959del (Exon 24)	Heterozygous		x with CNV				
25	c.2960‐18A>G	p.(Gly987Valfs*33)	r.2959_2960ins TCTCATTGTCTCTGCAG	Heterozygous		x		x	Affecting splicing	Benign
30	c.3559‐745A>G	p.(Arg1186_Val1187ins LysProArgLeuSerLys*)	r.3558_3559ins(94)	Heterozygous	x					
34	c.4102‐239A>G	p.(Gln1368Serfs*15)	r.4101_4102ins(89)	Heterozygous	x					
38	c.4405‐9T>G	p.(Val1469Ilefs*4)	r.4404_4405insATTTTCAG	Heterozygous		x		x	Affecting splicing	Disease causing

*Note*: The asterisk indicates that this mutation was only detected if present in heterozygous state. For Human Splicing Finder and MutationTaster open versions were used.^20^, ^21^

a If heterozygous, only detectable by NGS+CNV.

## 4 DISCUSSION

CPS1D is a rare metabolic condition with a nonspecific biochemical profile thus requiring additional tests.^5^ Nowadays, confirmation of the disease is usually done by molecular genetic investigation that led to the reporting of more than 230 *CPS1* mutations underlining the genetic heterogeneity at this locus.^7^, ^8^, ^9^, ^10^, ^11^, ^12^, ^13^, ^14^ In recent years, NGS became the preferred method for *CPS1* molecular genetic investigation either as part of (often custom‐made) gene panels or of whole exome or genome sequencing. In our laboratory, RNA sequencing was the preferred method in the past 15 years.^11^, ^16^ In this study, we investigated retrospectively the theoretical sensitivity if different sequencing approaches would have been used for detecting *CPS1* mutations, and, based on our findings, suggest a diagnostic algorithm for molecular genetic testing of CPS1D.

We based our analysis on 155 genetically confirmed cases comprising 172 different variants and 136 different genotypes. Main finding was that in CPS1D, private mutations are the rule, and in about 10% of the genotypes a complex mutation is present. Underlining the relevance of RNA sequencing, 7/14 complex mutations required for correct interpretation RNA analysis, and 5/14 complex mutations would have been missed if NGS was done without CNV analysis. In contrast, RNA sequencing had identified all mutations.

Almost all of the potentially missed variants are intronic substitutions, deletions, or insertions affecting splicing. For instance, the deep intronic change c.3559‐745A>G creates a novel donor splice site leading to an insertion of a 94 bp pseudoexon with a premature termination codon (p.[Arg1186_Val1187insLysProArgLeuSerLys*]). The same occurred in the case of c.4102‐239A>G (p.[Gln1368Serfs*15]). As both these mutations lie in deep intronic sequences, likely neither direct Sanger sequencing nor NGS (with or without CNV) would have detected them. In contrast, RNA analysis using phytohemagglutinin stimulated lymphocytes or fibroblasts did in fact identify these mutations. Other examples illustrating the same principle are summarized in Table 2. Some of these additional examples comprise deletions (c.622‐52_711+1416del, c.712‐430_766del, c.2895+429_2960‐281del), which would have only been detected if CNV analysis was added to NGS. RNA analysis is however also important in case of changes close to the exonic sequences. As shown for mutation c.622‐24A>G, while this change would have likely been identified by all sequencing methods (apart possibly from whole exome sequencing), correct interpretation as a splicing mutation was not offered by in silico prediction (Table 2).

We had previously shown that RNA analysis can substantially shorten the time to diagnosis in CPS1D.^16^ We add here an improved diagnostic yield as another benefit of performing RNA analysis. With these two advantages in mind, we propose to consider adding RNA sequencing in so far mutation‐negative patients with a strong suspicion for CPS1D. In addition, it needs to be remembered that RNA sequencing is a straightforward and easy to perform analysis requiring only basic sequencing facilities but not high‐throughput techniques rendering establishing and performing this method feasible in many places. Based on these findings and considerations, we propose the following diagnostic algorithm: if available, RNA sequencing can be the first line genetic test in suspected CPS1D; in all other cases, NGS together with CNV should be performed with the option of adding RNA sequencing in mutation‐negative patients with a strong suspicion for CPS1D or in order to correctly interpret unclear intronic alterations that could possibly affect splicing.

In summary, molecular genetic investigation for CPS1D remains challenging as the specific locus shows a high variability with most mutations being private. In addition, many intronic sequences of *CPS1* are likewise prone to changes that may be missed with NGS, even if CNV analysis is added. Unclear cases may therefore need to be investigated by RNA sequencing in addition, if this method is not used as the initial diagnostic procedure.

## Supporting information


**Table S1** Novel missense mutations (n = 31) in the *CPS1* gene

**Table S2** Novel nonsense mutations (n = 2), deletions (n = 12), duplications (n = 2), insertions (n = 1), delins (n = 1), and splice errors (n = 13) of the *CPS1* gene
