# Supplementary material for: Improvement of diagnostic yield in carbamoylphosphate synthetase 1 (CPS1) molecular genetic investigation by RNA sequencing
Source: JIMD Rep. 2020 Jan 9;52(1):28–34. doi: 10.1002/jmd2.12091 (PMC7052687; doi:10.1002/jmd2.12091)
Supplement: Supplementary file 1 — Table S1 Novel missense mutations (n = 31) in the CPS1 gene [file JMD2-52-28-s001.docx]

**Supp. Table S1:** Novel missense mutations (n=31) in the *CPS1* gene

| **Exon** | **Codon** | **Nucleotide** | **Protein** | **Comments** |
| --- | --- | --- | --- | --- |
| 2 | 66 | c.197C>T | p.(Ser66Phe) |  |
| 8 | 263 | c.788G>C | p.(Gly263Ala) | Homozygous, died as neonate |
| 9 | 291 | c.872T>C | p.(Phe291Ser) | Homozygous, died as neonate |
| 9 | 316 | c.946A>G | p.(Arg316Gly) |  |
| 11 | 383 | c.1147G>T | p.(Gly383Trp) | Neonatal, together with p.(Ser913Leu) |
| 14 | 469 | c.1405G>T | p.(Val469Phe) |  |
| 14 | 471 | c.1412C>T | p.(Thr471Ile) |  |
| 14 | 485 | c.1454T>G | p.(Leu485Arg) |  |
| 16 | 573 | c.1718C>A | p.(Ala573Glu) |  |
| 16 | 588 | c.1763C>T | p.(Ser588Phe) |  |
| 17 | 637 | c.1910T>C | p.(Val637Ala) | gnomAD MAF (C) = 0.00005, 0 homozygotes |
| 18 | 700 | c.2099A>T | p.(Gln700Leu) |  |
| 18 | 727 | c.2180C>T | p.(Ser727Leu) |  |
| 19 | 770 | c.2308G>T | p.(Val770Phe) |  |
| 19 | 772 | c.2314A>C | p.(Lys772Gln) |  |
| 19 | 776 | c.2328G>T | p.(Trp776Cys) |  |
| 20 | 815 | c.2445G>A | p.(Met815Ile) | Neonatal, together with p.(Arg1259*) |
| 25 | 1013 | c.3038T>A | p.(Val1013Glu) |  |
| 25 | 1023 | c.3069C>A | p.(Asp1023Glu) |  |
| 25 | 1031 | c.3092A>G | p.(Tyr1031Cys) |  |
| 25 | 1040 | c.3118A>T | p.(Ile1040Phe) |  |
| 26 | 1054 | c.3161T>C | p.(Ile1054Thr) |  |
| 27 | 1134 | c.3402G>C | p.(Leu1134Phe) |  |
| 30 | 1197 | c.3589G>A | p.(Glu1197Lys) |  |
| 31 | 1238 | c.3713G>A | p.(Gly1238Asp) |  |
| 32 | 1254 | c.3761T>G | p.(Ile1254Ser) |  |
| 32 | 1262 | c.3784C>G | p.(Arg1262Gly) |  |
| 32 | 1263 | c.3788C>A | p.(Ser1263Tyr) |  |
| 33 | 1310 | c.3929C>A | p.(Ala1310Asp) |  |
| 33 | 1320 | c.3959A>T | p.(Asp1320Val) |  |
| 38 | 1484 | c.4451A>T | p.(Asp1484Val) |  |

All listed missense mutations were tested *in silico* with MutationTaster, PolyPhen2 and SIFT and were at least 2 times classified as “disease causing”. If not stated in comments, missense mutations were not found in the gnomAD database. CPS1 reference sequence: Ensembl ENSG00000021826, ENST00000233072.9; GenBank NG_008285.1, NM_001875.4
