# Supplementary material for: Improvement of diagnostic yield in carbamoylphosphate synthetase 1 (CPS1) molecular genetic investigation by RNA sequencing
Source: JIMD Rep. 2020 Jan 9;52(1):28–34. doi: 10.1002/jmd2.12091 (PMC7052687; doi:10.1002/jmd2.12091)
Supplement: Supplementary file 2 — Table S2 Novel nonsense mutations (n = 2), deletions (n = 12), duplications (n = 2), insertions (n = 1), delins (n = 1), and splice errors (n = 13) of the CPS1 gene [file JMD2-52-28-s002.docx]

**Supp. Table S2:** Novel nonsense mutations (n=2), deletions (n=12), duplications (n=2), insertions (n=1), delins (n=1), and splice errors (n=13) of the *CPS1* gene

| **Nonsense** | | | | |
| --- | --- | --- | --- | --- |
| **Exon** | **Codon** | **Nucleotide** | **Protein** | **RNA / Comments** |
| 27 | 1127 | c.3380T>A | p.(Leu1127*) |  |
| 32 | 1259 | c.3775A>T | p.(Arg1259*) | Neonatal, together with p.(Met815Ile) |
| **Deletions/Insertions/Duplications/INDELs** | | | | |
| **Exon** | **Codon** | **Nucleotide** | **Protein** | **RNA / Comments** |
| 2 | 43 | c.127-26_127-24delinsCAG | p.(Ala43Aspfs*22) | r.127_128ins(23) |
| 2 | 73 | c.210_217dup | p.(Phe73*) |  |
| 7 | 208 | c.622-52_711+1416del | p.(Asp208_Lys237del) | r.622_711del (Exon7) |
| 8 | 238 | c.712-430_766del | p.(Arg238Metfs*5) | r.712_840del (Exon 8) |
| 9 | 299 | c.850del | p.(Ser284Valfs*15) |  |
| 14 | 475 | c.1424del | p.(Gly475Alafs*2) |  |
| 15-19 | 518 | c.1549+124_2391+800del | p.(Val518Hisfs*8) | r.1550_2391del (Exon 15-19) |
| 16 | 604 | c.1812_1813del | p.(Glu604Aspfs*31) |  |
| 17 | 645 | c.1933_1940del | p.(Val645Leufs*2) |  |
| 18 | 685 | c.2055_2058del | p.(Ile685Metfs*30) |  |
| 18 | 694 | c.2079_2080ins  CATTCATTCATTCATT | p.(Val694Hisfs*8) |  |
| 18 | 724 | c.2171_2175del | p.(Arg724Glyfs*26) |  |
| 24 | 966 | c.2895+429_2960-281del | p.(Glu966Alafs*27) | r.2896_2959del (Exon24) |
| 32 | 1269 | c.3806del | p.(Lys1269Argfs*14) |  |
| 32 | 1304 | c.3910_3913del | p.(Asp1304Metfs*14) |  |
| 33 | 1312 | c.3935dup | p.(Met1312Ilefs*11) |  |
| **Splice Errors** | | | | |
| **Exon** | **Codon** | **Nucleotide** | **Protein** | **RNA / Comments** |
| 2 | 80 | c.236+1G>A | p.(Tyr80*) | r.236_237ins(71) |
| 5 | 177 | c.528+1G>T | p.(Gly177Leufs*2) | r.528_529ins(39) |
| 7 | 208 | c.622-24A>G | p.(Asp208_Lys237del) | r.622_711del (Exon7) |
| 9 | 316 | c.947G>T (last bp of exon 9) | p.(Arg316Metfs*2) | r.946_947insTGTGA |
| 16 | 570 | c.1708-3C>G | p.(Ile570_Lys612del) | r.1708_1836del (Exon16) |
| 24 | 987 | c.2960-18A>G | p.(Gly987Valfs*33) | r.2959_2960insTCTCATTGTCTCTGCAG |
| 30 | 1186 | c.3559-745A>G | p.(Arg1186_Val1187ins  LysProArgLeuSerLys*) | r.3558_3559ins(94) |
| 32 | 1282 | c.3927+2T>A | p.(Val1282_Lys1309del) | r.3844_3927del |
| 33 | 1309 | c.3928-4A>G | p.(Lys1309_Ala1310insGln) | r.3927_3928insCAG |
| 33 | 1328 | c.4002+1G>A | p.(Glu1328Glyfs*5) | r.3980_4002del |
| 35 | 1368 | c.4102-1G>A | p.(Gln1368_Lys1387del) | r.4102_4161del (Exon 35) |
| 36 | 1388 | c.4162-2A>G | p.(Leu1388Ilefs*3) | r.4162_4274del (Exon 36) |
| 37 | 1426 | c.4404+3A>G | p.(Leu1426*) | r.4275_4404del (Exon37) |
